# The Effect of Al and Ti Additions on Solid Solution Strengthening and Precipitation Hardening in CoNiFe Medium-Entropy Alloys

**DOI:** 10.3390/ma16186297

**Published:** 2023-09-20

**Authors:** Piotr Bała, Kamil Górecki, Rafał Dziurka, Tomasz Kozieł

**Affiliations:** 1 Faculty of Metals Engineering and Industrial Computer Science, AGH University of Krakow, Al. A. Mickiewicza 30, 30-059 Krakow, Poland; dziurka@agh.edu.pl (R.D.); tkoziel@agh.edu.pl (T.K.); 2 Academic Centre for Materials and Nanotechnology, AGH University of Krakow, Al. A. Mickiewicza 30, 30-059 Krakow, Poland; 3 Institute of Environmental Technology, Centre of Energy and Environmental Technology, 17. Listopadu 2172/15, 708 00 Ostrava, Czech Republic; kamil.maciej.gorecki@gmail.com

**Keywords:** high-entropy alloys, heat treatment, aging, microstructure, mechanical properties

## Abstract

The effect of Al and Ti additions on the microstructure and properties of CoNiFe alloys was studied in this paper. The investigations were conducted on four specially designed and produced arc furnace alloys (from 3 to 5 components, with medium to high entropy). Samples in various states were analyzed, i.e., as-cast, after homogenization, after solution heat treatment, and after solution heat treatment and aging. The obtained samples were characterized by: SEM observations, EDS, XRD, TEM analyses, and finally, hardness measurements. The solid solution strengthening coming from the addition of 5 at. pct. Al was negligible, while the effect from the 5 at. pct. of Ti addition was significant. The precipitation hardening effect related to the presence of the (CoNi)_3_Ti phase caused by the Ti addition is comparable with the total effect of the Al and Ti addition, which caused the precipitation of (NiCo)_3_AlTi.

## 1. Introduction

Metals and their alloys are frequently chosen for structural applications as they have a desirable combination of mechanical properties. Single-phase alloys can be strengthened by grain size reduction, solid-solution alloying, and strain hardening. Multi-phase alloys can be additionally strengthened by precipitation hardening. All strengthening mechanisms rely on restricting or hindering dislocation motion, rendering a material stronger and harder. The mechanism that plays the most relevant role depends on the selected application. The final properties of alloys are often the result of not one, but two or more strengthening mechanisms [1,2].

High-entropy alloys (HEAs) belong to a group of the most promising metallic materials in modern materials science and engineering. They represent a fundamental difference from metal alloys’ existing design, production, and development. Instead of starting with one base element, which accounts for most of the known alloy composition, and adding dilute amounts of other elements, HEAs focus on the unexplored central regions of multi-element phase diagrams, where three or more alloying elements occur in concentrated amounts, with no obvious base element. This means millions of new alloy systems.

So how to define HEAs? HEAs are composed of at least five elements with the contents of each element between 5 and 35 atomic pct. [3,4]—a composition-based definition. The entropy-based definition says that in HEAs the mixing entropy (ΔSmix) should be higher than 1.61 R (where R is the gas constant) for equimolar alloys [5]. This new group of materials is characterized by several so-called core effects: (a) high-entropy effect; (b) severe lattice distortion effect; (c) sluggish diffusion effect; (d) cocktail effect [4,5,6,7,8].

The field of HEAs offers new challenges in establishing relationships between composition, microstructure, and properties. According to the literature [9,10,11,12,13,14,15,16], HEAs may be characterized by high hardness, wear resistance, high-temperature creep resistance, and oxidation resistance. Initially (almost 20 years ago), the goal in HEAs was to achieve a simple solid solution, with face-centered cubic (FCC) or body-centered cubic (BCC) or both (FCC + BCC) structures. In practice, obtaining a solid solution in the materials’ entire volume is extremely difficult, as HEAs have a complex chemical composition [8]. Many chemical and thermodynamical factors have to be taken into account. Another important factor is the choice of synthesis method and heat treatment parameters, which define the materials’ state. The original view of HEA’s has recently changed. Materials with a high-entropy matrix and additional precipitates possess a vast application potential [17,18,19].

The goal of our research is to investigate the effect of Al and Ti additions on the solid solution strengthening and precipitation hardening in CoNiFe medium-entropy alloys. Ni, Co, and Fe dissolve into each other to form solid solutions. With the right additives, the alloy can be used at elevated temperatures, or if the FCC structure is obtained, it can operate at lowered temperatures. It is a very good starting point for the search for new alloys with unique properties [8,20].

## 2. Materials and Methods

We chose Co, Ni, and Fe as they dissolve in each other to form a solid solution and have similar metallic atomic radii (Co = 1.251; Ni = 1.246; Fe = 1.241 Å). If the Ni and Co contents are higher than that of iron (∆Hmix CoFe = −1 kJ/mol, ∆Hmix CoNi = 0 kJ/mol, and ∆Hmix FeNi = −2 kJ/mol), they form solutions with a face-centered cubic structure (FCC). As an alloying addition, we chose Al and Ti, as they have a similar atomic radius (1.432 Å for Al and 1.462 Å for Ti), larger than Co, Ni, and Fe, but they differ in crystal structures. Al has an FCC structure and Ti has a hexagonal structure (HCP). Moreover, Al and Ti are prone to creating intermetallic phases instead of a solid solution in this element system. Yang et al. presented in Ref. [21] the thermodynamic factors required for high-entropy alloys, namely, (i) mixing enthalpy ∆Hmix between −15 and −5 kJ/mol, (ii) the atomic ratio difference parameter δ ≤ 6.6 pct., and (iii) entropy to enthalpy ratio Ω ≥ 1.1. We designed and manufactured 4 alloys and their nominal chemical compositions are shown in Table 1. The full solubility of Co, Ni, and Fe is confirmed by thermodynamic factors determined based on the nominal composition of the M1 alloy: mixing enthalpy close to 0, low δ value resulting from similar atomic ratio (Hume–Rothery rule). The M2, M3, and M4 alloys fulfill the above-mentioned requirements (see Table 1); however, only the M4 alloy fulfills the composition-based HEA definition. The M1, M2, and M3 alloys can be classified as medium-entropy alloys. Additionally, the Valance Electron Concentration (VEC) proposed by Guo et al. [22] (when the VEC value is lower than 6.8—BCC structure, higher than 8—FCC structure, and between those values—BCC + FCC structure) was calculated, suggesting that all investigated alloys should have a face-centered cubic structure.

The alloy selection was followed by thermodynamic calculations using the Thermo-Calc software with a HEA-dedicated TCHEA5 database. This made it possible to formulate initial predictions about the melting temperatures and phase compositions of the studied HEAs. The Scheil solidification simulation was used as it enables obtaining information about non-equilibrium or partial-equilibrium transformations.

The alloys were prepared by the arc melting of a mixture of high-purity metals (purity higher than 99 wt%) on a water-cooled copper mold under a Ti-gettered argon atmosphere using Arc Melter AM (Edmund Bühler GmbH, Bodelshausen, Germany). We investigated our alloys in 4 states. After casting, we cut off a part of the ingots. Next, the rest of the ingots were annealed in a vacuum furnace for 20 h at 1100 °C with furnace cooling to obtain chemical homogeneity throughout the entire volume. Once again, we cut off a part of the ingots. The ingots were then supersaturated for 1 h at 1200 °C and subsequently water-quenched. We cut off a part of the ingots and the remaining material was aged at 700 °C for 4 h with air cooling. Heat treatment parameters were selected based on the results presented in our previous paper [23].

Microstructural investigations were conducted by means of light microscope (LM—Nikon Eclipse LV150N, Grand River Road Brighton, MI, USA), scanning electron microscopy (SEM—FEI Versa 3D, Hillsboro, OR, USA) equipped with an Oxford Instruments Ultim Max energy-dispersive X-ray (EDX, Abingdon, UK) spectrometer. The analyses were performed at an accelerating voltage of 20 kV spot size 3.0. Transmission electron microscopy (TEM) investigations were conducted on a Tecnai TF20 X-TWIN (FEG) microscope (FEI, Hillsboro, OR, USA) working at an accelerating voltage of 200 kV.

Samples for LM observations were prepared using standard metallographic techniques and polished using silica oxides with a small addition of malic acid. Next, the samples were etched using a 4 g CuSO_4_ + 20 mL HCl + 20 mL C_2_H_5_OH solution for about 2 s. Thin foils for TEM investigations were mechanically prepared and electropolished in a 10 pct solution of perchloric acid in methanol, at −12 °C and 20 V. Hardness measurements were performed by means of an automated hardness tester (Tukon 2500 Willson-Hardness, Canton, MA, USA) and Vickers indenter with the applied force of 9.81 N.

Phase composition was determined using X-ray powder diffraction (XRD) technique. XRD patterns were obtained using Rigaku SmartLab diffractometer (Rigaku, Tokyo, Japan) with detector D/teX Ultra 250. The source of X-ray irradiation was Co tube (CoKα, λ1 = 0.178892 nm, λ2 = 0.179278 nm) operated at 40 kV and 40 mA. Incident and diffracted beam optics were equipped with 5° Soller slits; incident slits were set up to irradiate the area of the sample 10 × 10 mm (automatic divergence slits) constantly. Slits on the diffracted beam were set up to the fixed values of 8 and 14 mm.

The samples were obtained from gently obtained shavings of alloys (base grain size was too large for appropriate XRD analysis) and placed on a rotational sample holder and measured in the reflection mode (Bragg–Brentano geometry). The samples were rotated (30 rpm) during the measurement to eliminate the preferred orientation effect. The XRD patterns were collected in a 2θ range of 5–90° with a step size of 0.01° and speed 0.5 deg.min^−1^. The measured XRD patterns were evaluated using PDXL 2 software (version 2.4.2.0) and compared with database PDF-2, release 2015 (ICDD, Newton Square, NC, USA).

## 3. Results

**Figure 1:**
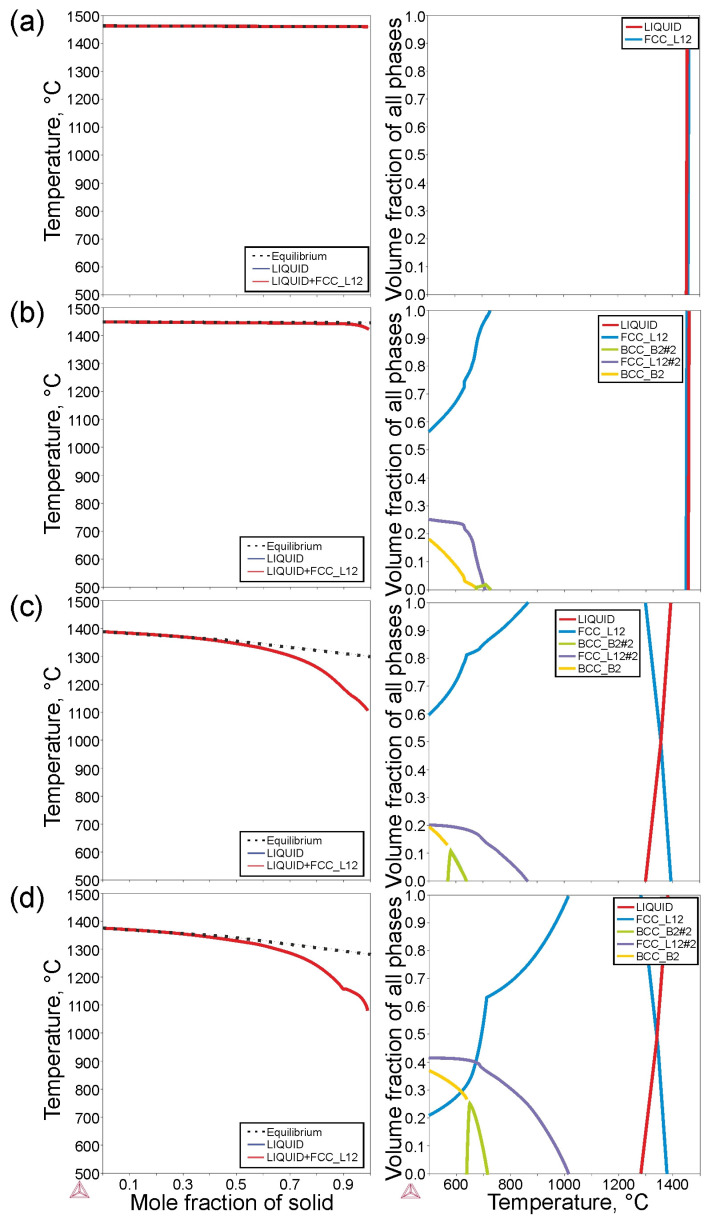
Thermodynamic calculations of the investigated alloys, Scheil non-equilibrium calculations (**left**), and Thermo-Calc equilibrium calculations (**right**) for: (**a**) M1, (**b**) M2, (**c**) M3, (**d**) M4 alloys.

The selected phase diagrams and matrix phase structures obtained from the Scheil solidification simulation and Thermo-Calc equilibrium calculations are shown in Figure 1. Only the FCC solid solution should be present in a 3-component alloy (M1). The calculated liquidus temperature of this alloy is 1459 °C. In the four-component alloy with the addition of Al (M2, Figure 1b), there should be a solid solution of FCC and BCC, and precipitation of the Ni_3_Al phase (γ′ phase). The addition of aluminum caused a slight decrease in the liquidus temperature to 1450 °C and an extension of the solidification range. The solidus temperature was 1426 °C. In the second four-component alloy with the addition of Ti (M3, Figure 1c), a significant reduction in the liquidus temperature to 1390 °C and the solidus temperature to 1107 °C was found. In this alloy, there should be two solid solutions of FCC and BCC and precipitates of the Ni_3_Ti phase (η phase) and an ordered phase with a BCC structure. In the five-component alloy, with the addition of Al and Ti, marked as M4 (Figure 1d), there should be two solid solutions of FCC and BCC, and precipitates of the Ni_3_Al phase (γ′ phase) and the ordered phase with the BCC structure.

**Figure 2:**
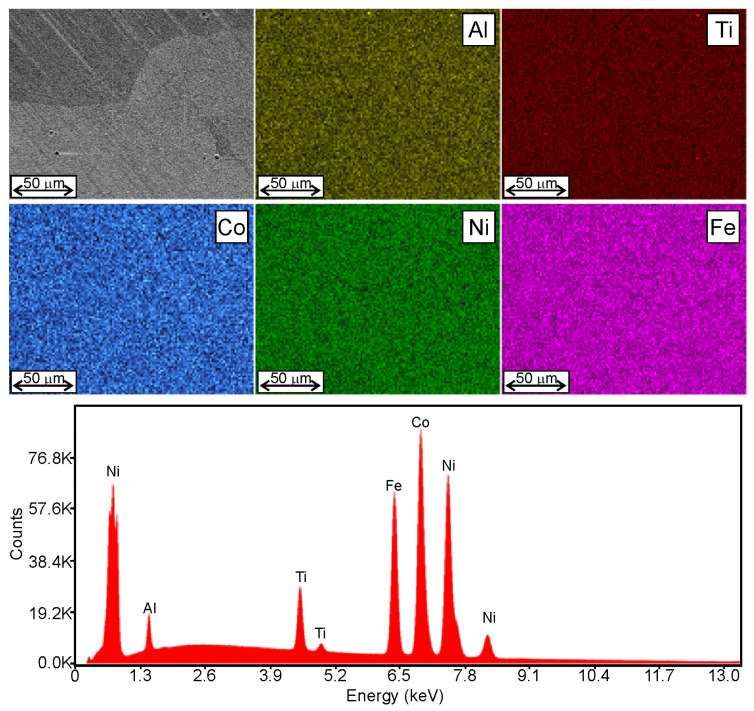
EDS map of the elements’ distribution and spectrum acquired from the analyzed area of the M4 alloy after annealing.

In our previous paper [24], we showed that, in HEAs in as-cast state, there can be large differences in the chemical composition of dendrites and interdendritic regions. These differences can be reduced by annealing, which we proved on the example of the M4 alloy, in which the elemental distribution after annealing was homogeneous (Figure 2). Therefore, after casting the alloys, they were annealed, and this was the state for further research, i.e., solution heat treatment and solution heat treatment followed by aging.

**Figure 3:**
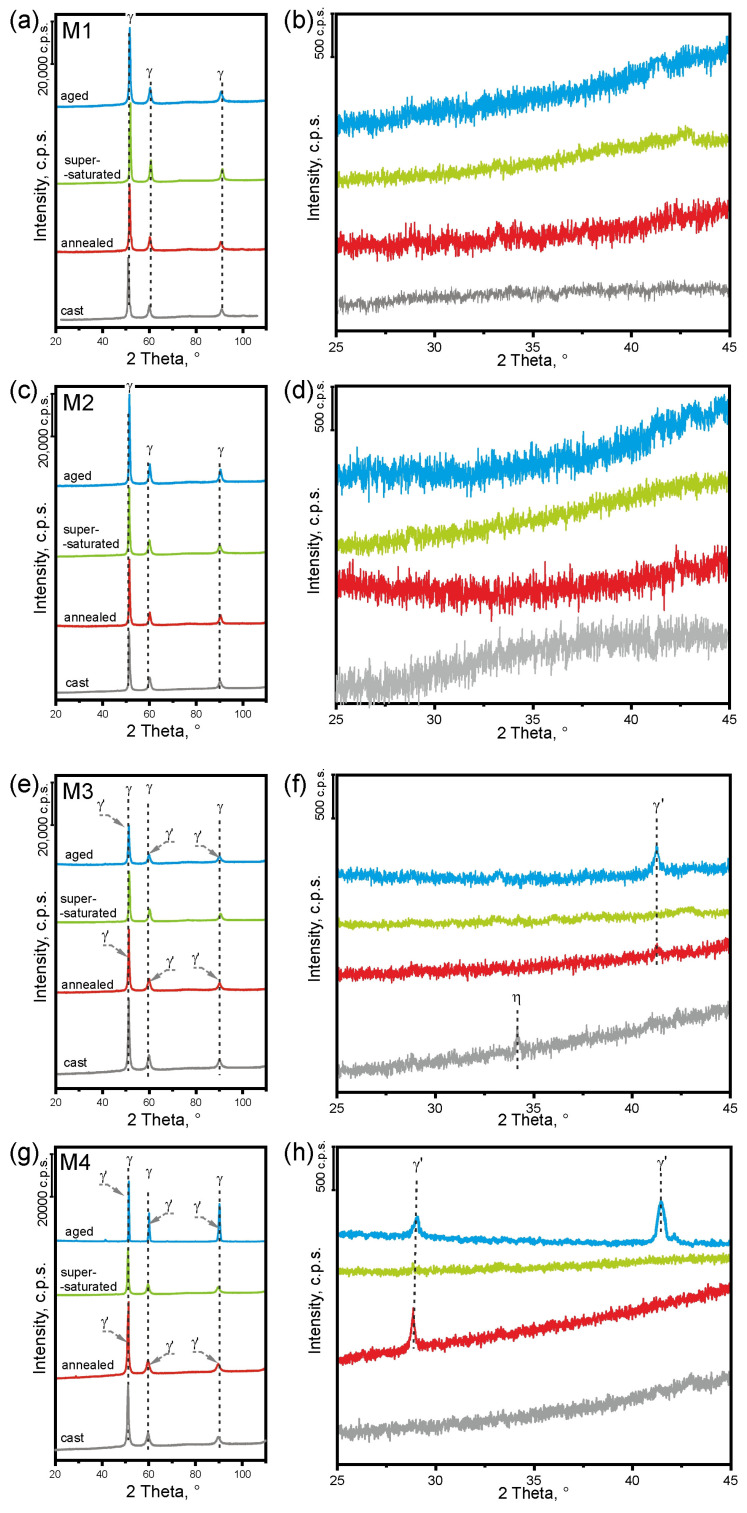
X-ray diffractograms of the investigated alloys in different conditions. (**a**,**b**) M1 alloy; (**c**,**d**) M2 alloy; (**e**,**f**) M3 alloy; (**g**,**h**) M4 alloy. (**b**,**d**,**f**,**h**) are the magnification of figures (**a**,**c**,**e**,**g**), respectively, at 2 Theta 25–45 to show peaks related to intermetallic phases. The meaning of the colors in the firgure: grey—as cast, red—annealed, green—supersaturated, blue—aged state.

XRD analysis was performed (Figure 3) in various states for all investigated alloys. It was confirmed that only the FCC solution is present in the M1 alloy (Figure 3a,b), regardless of the condition, similar to the M2 alloy (Figure 3c,d). In the M3 alloy (with the addition of titanium) in the as-cast state, the FCC solid solution and the η (Ni_3_Ti) phase were found (Figure 3e,f), consistent with the thermodynamic calculations presented above. This phase was dissolved after annealing. The (CoNi)_3_Ti phase appeared in its place (Figure 3f). Only the FCC solid solution was found in the state after solution heat treatment, which confirms the well-chosen parameters of the solution heat treatment process. After solution heat treatment followed by aging, the FCC solid solution and (CoNi)_3_Ti phase were found (Figure 3f—blue curve). In the M4 alloy (five-component), only the FCC solution was found in the state after casting and after solution heat treatment (Figure 3g,h). After annealing and solution heat treatment followed by aging, the FCC solid solution and γ′ (NiCo)_3_AlTi phase were found (Figure 3g,h—red curves). Table 2 shows the FCC lattice parameters determined in a supersaturated state. The increase in lattice parameters for the M2, M3, and M4 alloys compared to the M1 alloy resulted from the difference in the metallic radius size of the individual elements and, in the case of Ti, additionally from its different crystallographic structure. The results for the M2 and M3 alloys (respectively, with the addition of 5% Al and 5% Ti) show that the Al addition increased the lattice parameter of the solid solution more strongly. The partial substitution of Co, Ni, or Fe by aluminum and/or titanium is directly reflected in the size of the lattice parameters, which should affect the solution strengthening of the alloys.

**Figure 4:**
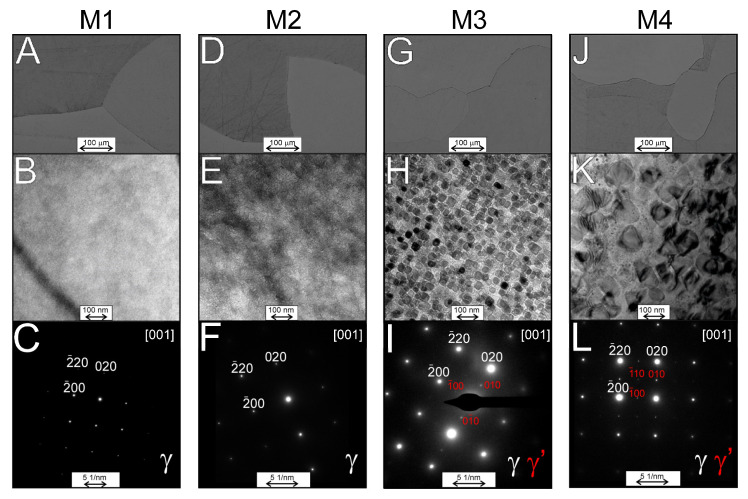
The microstructure of the investigated alloys after annealing: (**A**,**D**,**G**,**J**)—light microscope; (**B**,**E**,**H**,**K**)—bright-field TEM images; (**C**,**F**,**I**,**L**)—selective area diffraction patterns. (**H**) M3 alloy γ matrix with (CoNi)_3_Ti precipitates; (**K**) M4 alloy γ matrix with (NiCo)_3_AlTi precipitates.

To verify the XRD results, we decided to perform a detailed microstructure analysis of the investigated alloys in the annealed condition. It is the state closest to the state of thermodynamic equilibrium. After annealing, the investigated alloys were characterized by large grains (Figure 4A,D,G,J—LM), without visible precipitates. Changes caused by differences in the chemical compositions of the investigated alloys were observed in microstructures during TEM observations (Figure 4B,E,H,K). No precipitates were found in the M1 and M2 alloys, which was also confirmed by electron diffraction analysis (Figure 4C,F—for M1 and M2, respectively), in which only reflections corresponding to the γ phase were observed.

**Figure 5:**
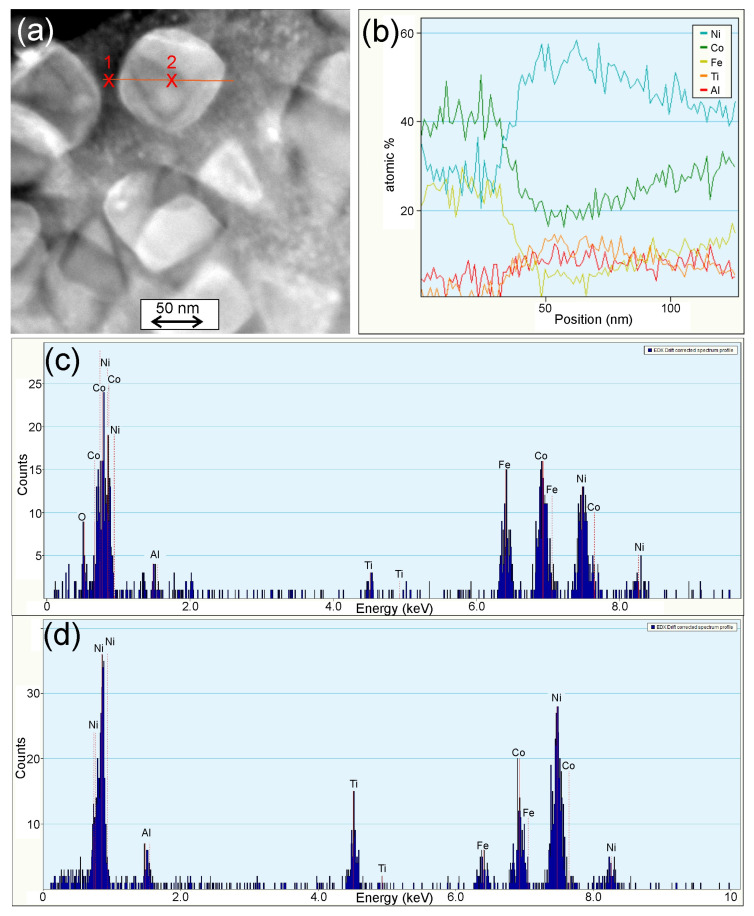
Line scan EDS analysis of the γ′ phase precipitate in the M4 alloy. (**a**) STEM image with marked analysis line, (**b**) change in chemical composition, (**c**) spectrum acquired from point 1—matrix, (**d**) spectrum acquired from point 2—precipitate.

In the M3 and M4 alloys, in addition to the γ matrix, small precipitates of (CoNi)_3_Ti and (NiCo)_3_TiAl in the M3 and M4 alloys, respectively, were found, as shown in Figure 4H and K and on selective area electron diffraction patterns (Figure 4I,L). The chemical composition of both above-mentioned γ′ phases were confirmed by EDS analysis (as an example for the M4 alloy in Figure 5). The precipitates observed for both alloys differed in shape and size. In the case of the M3 alloy, cuboid geometry precipitates were observed, with a size between 25 and 35 nm, while for the M4 alloy, the precipitates had an intermediate form between cuboid and spheroidal, and their size was between 50 and 60 nm.

**Figure 6:**
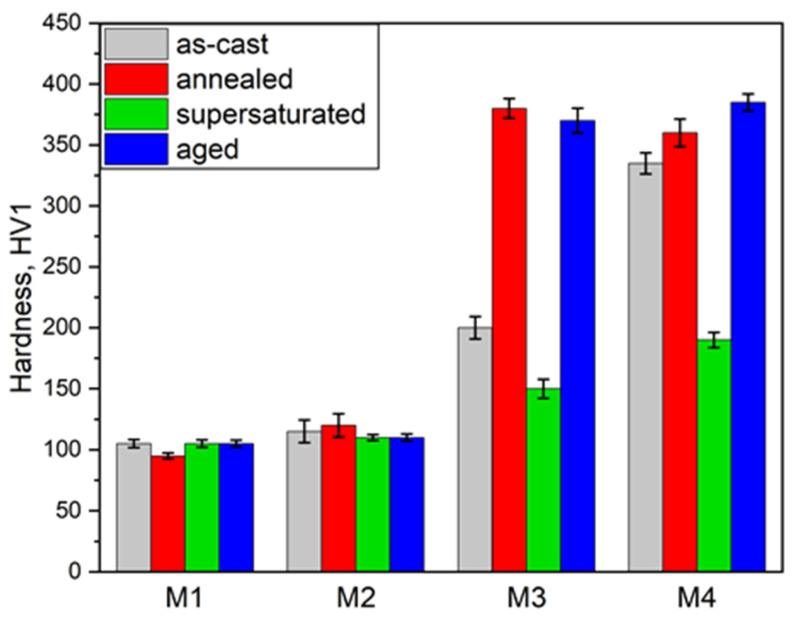
Hardness of the investigated alloys in different conditions.

The next stage of the study was to compare the hardness of the investigated alloys in different states, as shown in Figure 6. The hardness of the M1 alloy (105 HV1) is a typical value for the γ solution for the investigated Co-Ni-Fe system. The hardness of about 100 HV1, despite the lower hardness of the individual elements, results from the differences in the crystallographic structure of the individual elements (Co: HCP, Ni: FCC, Fe: BCC) and the differences in atomic bond strengths [25]. Regardless of the material state, the hardness of the M1 alloy is close to 100 HV1. The increase in the hardness of the M2 alloy (110 HV1), relative to the M1 alloy, is associated with additional solution strengthening caused by the addition of 5 at. pct Al. The M2 alloy has a similar hardness in all states of about 110 HV1.

In the case of the M3 alloy, the increase in hardness in the as-cast state compared to the M1 alloy (200 and 105 HV1, respectively) is associated with the addition of titanium, which also leads to solution strengthening, however, mainly with the probable precipitation of the γ′ phase. The phenomenon of the precipitation of the nanoscale intermetallic phases during crystallization in HEAs was experimentally confirmed by the authors of Ref. [26], who studied the CoCrCuMnNi alloy. This explains the high hardness of the M3 alloy after annealing (380 HV1), due to the precipitation of the γ′ phase. The strengthening resulting from the addition of titanium to the solid solution is significant and can be seen in the hardness of the M3 alloy after solution heat treatment, which is 150 HV1. This is 45 HV1 higher than the M1 alloy and 40 HV1 higher than the M2 alloy hardness. The hardness of the M3 alloy after solution heat treatment and aging is close to the hardness after annealing (370 HV1).

The hardness of the M4 alloy is similar to that of the M3 alloy, with the difference that the hardness of the M4 alloy in the as-cast state and after solution heat treatment is higher, 335 HV1 and 190 HV1, respectively. Most likely, the volume fraction of the γ′ phase precipitates was much larger in the M4 alloy in the as-cast state than in the M3 alloy. In the supersaturated state, however, the increase in hardness in comparison to the M3 alloy by 40 HV1 results from the combined strengthening of Al and Ti. Interestingly, after annealing and solution heat treatment and aging, the hardness of both alloys is similar. The hardness of Ni-Co-Fe alloys strengthened with γ′ phase precipitates depends largely on the chemical composition of the matrix and γ′ phase precipitates, the size and distribution of γ′ phase precipitates, and the lattice misfit parameter δ (δ = 2(aγ′ − aγ)/(aγ′ − aγ) [20].

## 4. Discussion

Four basic hardening mechanisms are used in polycrystalline materials: solid solution hardening, grain boundary hardening, dislocation hardening, and precipitation hardening. As the four mechanisms operate independently, the yield strength is a simple summation of the above-mentioned mechanisms. In our work, we addressed solid solution and precipitation hardening. However, traditional approaches to measuring the effect of solid solution strengthening are all based on dilute solution alloys, especially for binary systems [27,28,29]. For HEAs, the terms “solute” and “solvent” lose their conventional meanings. Therefore, we used the Ni-Co-Fe system, known for its mutually unlimited solubility, by designing the medium-entropy M1 alloy, to which we added Al and Ti in such a way as to obtain an increase in entropy. Still, at the same time, it was possible to obtain solid solution and precipitation strengthening. We conducted this because recent studies indicated that high-entropy alloys (HEAs) possess unusual structural and thermal features, which could greatly affect the dislocation motion and contribute to mechanical performance. However, a HEA matrix alone is insufficiently strong for engineering applications, and other strengthening mechanisms must be incorporated [30,31,32,33,34].

As expected, the alloy of Ni, Co, and Fe (M1), regardless of the state, is composed only of the FCC solid solution (Figure 3a,b). Despite thermodynamic predictions, the addition of 5% Al did not result in the formation of an additional BCC solid solution or precipitation of the γ′ phase, regardless of whether it was an annealed state or after full heat treatment (Figure 3c,d). The influence of Al on the solution strengthening, at 5 at.%, provides an increase in hardness of about 4.7%. The addition of 5% at. Ti (M3 alloy) caused an increase in hardness by 43%, although the addition of Ti did not cause such a large increase in the FCC lattice parameter as the addition of Al (see Table 2). In the M3 alloy, the η phase occurred after casting (Figure 3e,f—grey curves), and the (CoNi)_3_Ti phase occurred after annealing and full heat treatment (Figure 3e,f—red and blue curves respectively). This shows the possibility of precipitate strengthening in this alloy. The effect of precipitation strengthening is very large. It is most clearly seen in the annealed condition. The increase in hardness with respect to the M1 alloy is 357% (Figure 6). The observed (CoNi)_3_Ti phase is coherent with the matrix and, considering the small size of the precipitates, provides a strong increase in strength. Similar observations were made by He et al. [35], using small additions of Ti and Al to create nanoprecipitates, causing a strong increase in mechanical properties in the FeCoNiCr alloy.

Interestingly, in the five-component alloy (M4), where we added Ti and Al simultaneously, a clear increase in hardness (Figure 6) in the state after solution treatment can be seen (about 80% in relation to the M1 alloy). However, in the state after full heat treatment, the influence of both these elements (Ti and Al, M4) is similar to the addition of Ti (M3) alone. It indicates that the (CoNi)_3_Ti phase has a stronger precipitation effect than the (NiCo)_3_AlTi phase. It is known that the type of the precipitated phase and the size and shape of the precipitates are crucial for the mechanical properties of the conventional alloys. The shape of the precipitates is closely related to the lattice misfit δ between the precipitated phase and the matrix [29,30,31], such as in Ni-based superalloys [20], in which the cuboidal-ordered nanoparticles with an L12 structure (Ni_3_Al-type) are coherently embedded into the disordered FCC solid solution matrix, exhibiting an incomparable mechanical property, especially at high temperatures. Such precipitates were observed in the M3 alloy. The Ti addition effect confirms the data presented by Roth et al. [29].

To estimate the increase in the yield strength of the investigated alloys, resulting from the addition of Ti and Al, we used the method proposed by Gypen and Deruyttere [36,37]:

(1) $$∆\sigma =(\sum_{i}k_{i}^{l/n}c_{i}{)}^{n}$$

where n is a constant, c_i_ is the concentration of solute i, and k_i_ is the strengthening constant for solute i. Theoretical treatments indicate n could equal 2/3 or 1/2. This method has been found suitable to predict the yield strength of solid solution alloys for a series of experimental and commercial nickel-based alloys [29]. We obtained the coefficients for Ti and Al from [29]. The results of the calculations are presented in Table 3. The obtained results correlate very well with our observations. A single addition of Ti provides a very strong increase in the yield strength, comparable to the simultaneous addition of Ti and Al. Interestingly, we calculated the yield strength for an equimolar alloy composed of NiCoFeAlTi. Assuming a solid solution could be achieved in such an alloy, the yield strength would be slightly higher than in the M3 or M4 alloy, i.e., 353 MPa. As we showed in our previous work [24], producing such an alloy with a solid solution structure is impossible. A system of two disordered solid solutions of FCC and BCC, with numerous precipitates of brittle intermetallic phases, was formed. Such a microstructure does not allow additional strengthening by plastic deformations or grain refinement. The achieved microstructure of the disordered FCC solid solution matrix offers such possibilities. It is also worth mentioning that metals and alloys with an FCC crystallographic structure do not show a brittle transition temperature, which is important for materials operating below ambient temperature. On the other hand, the possibility of dissolving and precipitating reinforced intermetallic phases (such as (CoNi)_3_Ti in the M3 alloy) can influence the morphology of their precipitates, which translates into the effect of precipitation hardening. Such materials can work at elevated temperatures. Design strategies for new high-entropy alloys can be different. For example, very interesting results were obtained in alloys with a dominant FCC matrix and small volume fractions of another BCC or HCP solid solution and precipitates of intermetallic phases [30,35,38]. The expected properties and the predicted applications of new alloys determine which strengthening mechanisms should be chosen.

## 5. Conclusions

The addition of elements with similar metallic atomic radii but different crystallographic structures (Al and Ti) to the Co-Ni-Fe alloy caused higher solution strengthening by the element with a different crystallographic structure than the solvent. The solid solution strengthening coming from addition of 5 at. pct Al to the Co-Ni-Fe alloy is negligible, while the addition of 5 at. pct of Ti significantly increases the solid solution strengthening. The precipitation hardening effect related to the presence of (CoNi)_3_Ti phase caused by the addition of 5 at. pct Ti is comparable with the total effect of the 5 at. pct Al and 5 at. pct Ti addition, which caused the precipitation of γ′ ((NiCo)_3_AlTi). Precipitation hardening is much greater in the investigated alloys than the solid solution hardening.

We showed a successful way for effectively strengthening the FCC-HEA system (NiCoFe) using a small Ti addition. The microstructure obtained in the M3 alloy consists of precipitates with an L12 structure and structurally coherent with FCC matrix. We showed one of the ways of searching for new high-entropy alloys.

## Figures and Tables

**Table 1:** Nominal chemical compositions, thermodynamic parameters, and VEC of the investigated alloys.

	Al	Ti	Co	Ni	Fe	ΔH_mix_, kJ mol^−1^	ΔS_mix_, J mol^−1^ K^−1^	δ, pct.	Ω, -	VEC, -	Predicted Structure
M1	0	0	39	39	22	−1.03	8.88	0.30	15.18	9.17	FCC
M2	5	0	37	37	21	−4.43	10.09	3.22	3.91	8.86	FCC
M3	0	5	37	37	21	−6.31	10.09	3.74	2.82	8.91	FCC
M4	5	5	35	35	20	−9.54	11.27	4.75	2.04	8.6	FCC

**Table 2:** Lattice parameters for the investigated alloys estimated from the XRD analysis in the solution heat treated state, in nm.

M1	0.354367 (4)
M2	0.35771 (2)
M3	0.35636 (2)
M4	0.35904 (2)

**Table 3:** The yield strength for the investigated alloys calculated by the method proposed by Gypen and Deruyttere.

Pure Ni	110 MPa [20]
M1	186 MPa
M2	200 MPa
M3	298 MPa
M4	304 MPa
Equimolar NiCoFeAlTi alloy	353 MPa

## Data Availability

Data are available upon request.
